# “Where are we headed?” To better understand the career paths and barriers psychiatrists, psychologists, and psychotherapists face in Hungary. An outline of a quantitative and qualitative study

**DOI:** 10.1192/j.eurpsy.2024.1661

**Published:** 2024-08-27

**Authors:** I. Bitter, B. Péley, M. Bérdi, B. Henrietta, K. Farkas, E. Gergics, P. Nagy, K. Pál, B. Ungvári, P. Szabó, I. Tiringer, M. Fülöp, G. Szőnyi

**Affiliations:** ^1^Department of Psychiatry and Psychotherapy, Semmelweis University, Budapest; ^2^Insitute of Psychology, University of Pécs, Pécs; ^3^Department of Crisis Intervention and Psychiatry, Péterfy Sándor Street Hospital-Clinic; ^4^ MentalPort Institute of Psychoanalytic Methods; ^5^National Institute of Menthal Health, Neurology and Neurosurgery - Nyírő Gyula Center; ^6^Roska Tamás Doctoral School of Sciences and Technology, Pázmány Péter Catholic University, Budapest; ^7^Department of Behavioural Sciences, University of Pécs, Pécs; ^8^Research Centre for Natural Sciences, Institute of Cognitive Neuroscience and Psychology; ^9^Karoli Gaspar University of the Reformed Church, Budapest, Hungary

## Abstract

**Introduction:**

Becoming a psychiatrist, clinical psychologist or psychotherapist involves a complex set of skills that require extensive training. Clinical practice development and professional and personal identity formation are closely intertwined and continue throughout one’s career. Individual and environmental factors influence dropout. The beginning stages of training are incredibly challenging for trainees and can be a time of vulnerability as they face early professional hurdles. We propose that certain educational factors, such as inadequate practical training and insufficient emotional support during professional dilemmas, play a crucial role in manifesting burnout or other symptoms, potentially leading to stagnation in one’s career.

**Objectives:**

The main objective of our study is to identify causes of disruption and/or discontinuation of the training/residency programs in psychiatry, clinical psychology, and psychotherapy. Our study also aims to highlight the causes of chronic exhaustion among trainees in mental health professions.

**Methods:**

The research team has developed a comprehensive questionnaire including two validated psychometric scales, the Effort-Reward Imbalance Questionnaire (ERI, Siegrist *et al.* Soc Sci Med 2004; 58 1483-99, Salavecz *et al.* J Men Psychosom 2006; 7 231–246) and the Mental Health Test (MHT, Vargha *et al.* J Men Psychosom 2020; 21 281–322). A quantitative analysis (Braun *et al.* Qual. Res. Psychol. 2006; 3 77–101) will be performed on the responses, following which interviews will be conducted with previous volunteers who participated in the study. The interviews will be evaluated through content analysis. Our survey is prepared with the involvement of all significant training centers in Hungary. The study was approved by the United Ethical Review Committee for Research in Psychology (EPKEB, approval numbers: 2021-109, 2023-101).

**Results:**

The participants’ main characteristics and the questionnaires’ results will be summarized with standard statistical methods, while the interviews will be analyzed with the help of qualitative methods.

**Conclusions:**

Based on the results of the described study, we aim to investigate the educational system’s impact on the career development and commitment of psychiatrists, psychologists, and psychotherapists in Hungary. Additionally, the research will yield valuable perspectives on how these factors affect the mental well-being of these professionals. Ultimately, the results could help address areas of concern and improve mental health professionals’ training.

* Presenting author

** The two authors contributed equally.

**Disclosure of Interest:**

None Declared

